# Lecture on the Importance of the Application of Physiology to the Practice of Medicine and Surgery

**Published:** 1865-11

**Authors:** E. Brown-Sequard


### SELECTED.

### LECTURE ON THE IMPORTANCE OT THE APPLICATION OF PHYSIOLOGY TO THE PRACTICE OF MEDICINE AND SURGERY.

By E. BROWN-SEQUARD, M. D.

This extremely interesting lecture was delivered before the Medical Society of the College of Physicians of Dublin. The aim of the author is to show that by the knowledge derived from experiments on animals, as well as that derived from other physiological researches, from the knowledge we derive from microscopic anatomy, and even from simple descriptive anatomy—especially as regards the base of the brain—combined with the careful study of pathological cases at the bedside, may be drawn a great many conclusions of importance to the practice of medicine, and we may be led to form a sure diagnosis in many otherwise obscure cases.

The lecture is so condensed that it is impossible to do justice to it by any abstract; and the subject is so interesting, and is treated in so masterly a manner, that our readers will be pleased to have laid before them the main portion of the text.

The author observes: "A complete revolution has been made in the practice of medicine within the present century by the study of experimental facts observed upon living animals; and if, together with this source of knowledge and that which springs from the comparison of those facts with morbid cases in our own species, we make an appeal to the other branches of physiology and also to normal anatomy, especially that of the nervous centres and of the nerves at the base of the brain, we shall find that to understand the symptoms of a large class of pathological cases in our own species becomes almost as easy as to read the alphabet. In fact, many of the most complicated, the most obscure, and the most unintelligible cases of nervous disease are as easily understood as the simplest case of bronchitis, or any other simple affection of the lungs, the bowels, &c., if we have the advantage of the light which experimental physiology and the anatomy of the base of the brain now afford.

"Suppose, for instance, a patient comes to us suffering from paralysis, an absolutely complete paralysis of the motion of one-half—say the right half—of the body, from the neck downwards. Suppose that, in addition to this paralysis of motion, he has also, on the same side, extreme hyperæsthesia or increase of sensibility in all those parts which are struck with the paralysis of motion. Suppose that we find not only extreme sensibility to touch—a symptom which we may measure accurately with a pair of compasses or the æsthesiometer—but also an extreme sensitiveness to tickling—a sensibility, by the way, quite distinct from the other. Let us suppose that we find, besides all this, that the sensibility to a prick or a pinch —in fact the sensibility to painful impressions of every kind—likewise the sensitiveness to changes of temperature—cold and heat—is also much increased. Thus you have these four species of sensibility—each of which—I repeat, is entirely distinct from the others—all greatly increased in that limb, which, at the same time, is apparently dead, inasmuch as it does not possess the least power of motion. Suppose further, that in the limbs and part of the trunk in that same side of the body the temperature is found greatly increased, that the circulation of the blood in that side is more energetic, the arteries being *fuller;* or, in other words, that there is decidedly more blood there than elsewhere in the body.

"All these symptoms, observe, belong to one side of the body—*the right.* If, now, we compare them with what we find on the opposite side, they acquire still greater interest. On examining the *left* side of the body, we find an absolutely reverse condition. We find that all the four species of sensibility of which I have spoken are lost—absolutely gone—on that left side. We find that there is, on that last side, (in opposition to the state which exists on the right side,) a complete power of motion, not the slightest diminution of the power of the will.

"Suppose we go further, and inquire into the condition of the *fifth* kind of sensibility (assuming that their number is only five); if we look for the special sensibility existing in the *muscles*, that sensibility which serves to the direction of our movements, we find that this peculiar sensibility remains perfect in the limbs of that left side, and that the motions of weight, of resistence, &c., derived from the muscular sense, also remain perfect in those otherwise anæsthetic parts. There is, therefore, on the *left* side a complete anæsthesia of the four first kinds of sensibility of which I have spoken, notwithstanding the persistence of the muscular sense; while on the *right* side the condition is exactly the reverse—the muscular sense is gone, and the other four species of sensibility exist in a greatly increased degree.

"But what about the degree of heat in the limbs of the left side, and what of the circulation? Here, too, the condition of things is reversed; for on the left side there is not only a degree of heat much inferior to that on the right side of the body, but there is also an actual diminution of heat if you compare the state of those parts with their normal condition; in other words, there is an *absolute* not simply a *relative* diminution of heat on the left side. Thus, on the right side, the temperature of the body has increased, while on the left it has diminished. The case is similar as regards the circulation, which is less full in the left side than in a normal state.

"These features, I think you will agree with me in saying, are striking enough; yet there will be many others in the same individual, upon which I cannot now dwell, but which are fully as interesting and equally difficult of explanation by the practitioner who is not perfectly *au courant* with the present state of physiological science.

"In *the face* (for example), on the side of the injury (admitting that an injury is the cause of these symptoms), there will be an increase of heat, an increase of sensibility, a contraction of the pupil, and a degree of occlusion of the eyelids; so that the eyes of the patient—if you look at them at the same time while open—are quite different one from the other. The eye on the side of the increased heat and hyperæsthesia seems smaller, because the opening of the eyelids is smaller than on the other side.

"All these effects we can produce in animals very easily: and it has been (in some respects) my good fortune to find a number of such cases in our own species. One of the most striking of them I saw at the London Hospital, in company with my dear and talented friend, Dr. Robert M'Donnell; in that case the various symptoms which I have described were as marved as possible.

"Now, what was the injury which produced all these remarkable effects? It was simply this—a complete transversal division of the right lateral half of the spinal cord in the neck; not simply a part, but, I repeat, the entire lateral half, *i. e.*, the posterior column, the lateral column, the anterior column, and the gray matter of that side had been divided transversely and completely. Owing to that injury all those symptoms existed.

"Now, I ask you, if any physician at the beginning of this century—not having the light afforded by the present state of physiological science, no matter how learned and able in other respects—had such a good living problem been presented to him, would have been able to understand such a case? Decidedly not. Nay, more, he would, in all probability, not have seen the case as I have described it. He would not have recognized the existence of some of the symptoms. He would likely have fallen into the same error as was committed by the great French Surgeon, Boyer, who had such a case, bvt who never found that tha sensibility was lost on the side still under the power of the will, until the nurse, who discovered the fact by a mere chance, told him of it.

"From such facts you will see the great importance of a thorough knowledge of physiology. The physiologist can have no difficulty in understanding such a case, for when he knows that the spinal cord is the organ conveying the orders of the will to the muscles—that the nerve-fibres, serving for voluntary movement, proceed along the spinal cord, so that those which serve for the movement of the limbs on the right side of the body pass along the right side of the cord, while those serving for movement of the limbs on the *left* side of the body pass along the *left* side of the cord; it is quite evident to him that such a division of the cord as I have described will cause loss of motion on one side and not upon the the other. Again, the sensitive nerve-fibres which serve to the four first kinds of sensibility of which I have spoken, proceed in the spinal cord in such a manner as to go into the *opposite* side of that organ from that side of the body from which they convey the sensitive impressions—so that the nerve fibres of sensibility in my right arm and right leg, for instance, pass into the left side of my spinal cord, and *vice versa*. Hence a division of the cord produces loss of sensibility on the side of the body opposite to that of the injury.

"Equally simple is the explanation of the increase of heat in the limbs on the side of the injury. The nerves of bloodvessels pass into the spinal cord on the side corresponding to that of the limbs into which they go, just as it is the case for the nerves of voluntary movement, so that a division of the spinal cord on the right side produces paralysis of the nerves of bloodvessels in the right side of the body, in consequence of which, the impulse of the heart being less resisted on that side than on the other, there is a great afflux of blood, and also, as an effect of this increased quantity of blood, an increased quantity of blood, an increased heat, and (as a consequence of both the increased heat and the augmented quantity of blood) in a measure, also the increased sensibility of which I have spoken—the hyperæsthesia of the four kinds of sensibility.

"I cannot further dwell on this class of cases. Sufficient has been said, I hope, to show how important the light physiology can throw on symptoms which certainly would have been most obscure (to say the least) to even the most eminent and learned men of the beginning of this century, who did not know the physiological facts which have since been discovered.

"I will now produce another case. Let us suppose a man has sustained an injury, not of the half of the spinal cord, but an injury of one-half of the medulla oblongata at the level of the decussation of the anterior pyramids; not such an injury as would destroy life at once, but an injury, a tumor, or a morbid alteration of sufficient extent to produce decided symptoms. In this case you would have all the symptoms which I have described in the former case, but with this difference, that as the anterior pyramids decussate there, an injury on the right half of the medulla oblongata would strike the fibres of voluntary movement belonging to that side previous to their making their decussation, or, in other words, before they pass from the right side into the left side; and it would also strike these fibres of voluntary movement that have come from the left side and have already made their decussation. In this case, therefore, there would be paralysis of motion on *both* sides of the body, while, as regards the state of the bloodvessels, the hyperæsthesia of the various kinds of sensibility, everything would be the same as in the former case.

"Let us take, now, a case of injury a little higher up, and we shall find other striking differences.

"A patient, I suppose, comes to you with paralysis of the external rectus on the eye of the right side. The face is also paralyzed on the same side. There is, besides, anæsthesia of the face on that side; while the left side of the body is affected with paralysis both of sensibility and motion. Here there is a case absolutely distinct from both the others. I cannot dwell, at length, on the remaining symptoms of the case, but I must not pass from it without noticing one striking feature. You will often find, in such cases, that the tongue of the patient is perfectly free; there is no loss of movement at all in that organ, and there is no impediment of speech. You will find shat the facial paralysis is of exactly the same kind as that which takes place when the facial nerve, outside of the cranium, has been injured—*i. e.*, the muscles which communicate expression to the face and the orbicularis palpebræ are paralyzed. This case, therefore, is quite distinct from cases of hemiplegia arising from disease of the brain. As you are aware, in cases of paralysis arising from injury to the brain, the paralysis of the face is on the same side with that of the body, and the orbicularis is not paralyzed, while the tongue is almost always somewhat paralyzed. In the case I am now speaking of, the distinction is striking. The drawing of the face on one side, owing to the paralysis of the other side, takes place on the side of the paralysis of the body instead of on the other side—because the paralyzed side of the face is the opposite to that which is usually paralyzed. Besides all this you will find that the sense of taste is altered in a good part of t ie tongue, on the side at which the face is anæsthetic. You will find further that the patient is in a state of considerable emotion—he will shed tears and cry easily; he w ll gape frequently, and while gaping there will frequently be a sudden jerk of the paralyzed limbs. There is also, generally, considerable giddiness and tendency to vomit. I am now mentioning only the principal symptoms.

"Now, I ask, what is the explanation of this case? Do you think that the most eminent men of the beginning of this century, not knowing the sciences of physiology and anatomy as we now know them, could have understood this case? Certainly not; and thus, you perceive, an acquaintance with physiology and anatomy is an immense help in the diagnosis of disease.

"The series of symptoms I have last described belong to a case of injury of the *pons Varolii*, striking, at the same time, one side of the trigeminal and of the facial nerves before they have made their decussation, which is at the lower part of the pons Varolii, and striking, · lso, one side of the sixth pair of nerves before it has made its decussation with its fellow of the other side, thus producing paralysis on the side of the injury just as if the injury existed in the nerve itself. You must not, however, suppose that an alteration of the pons Varolii will, under all cir umstances, produce these effects. If the injury takes place a little higher up than the lower part of the organ, striking at the place where the facial nerve and part of the trigeminal nerve make their crossing, you will have these results; both sides of the face will be paralyzed, as regards sensibility and motion, together with the action of the external recti of the eye, and also the sense of taste in the anterior part of the tongue, while the paralysis of sensibility and motion in the body will be only on one side. In order to understand this it is quite sufficient to keep in view what the nerves of the face do when they reach the pons Varolii. When the injury strikes that nervous centre above the decussation, you will have, so far as regards the portions of the face and body which are paralyzed, the same effects as are observable in most cases of brain disease, viz., the paralysis of the face will be on the same side with the paralysis of the body. If, on the other hand, as already stated, the injury strikes the pons Varolii below the decussation, the opposite effects are produced.

"The question arises, therefore, how can you know whether the seat of the disease is in the pons Varolii if you have not that peculiar symptom of the difference of the sides, as regards the paralysis of the face and that of the body? There are several peculiar features, which I cannot dwell upon, which will answer that question. In the first place, if the injury is in the pons Varolii, you will find in the beginning of the affection one most important symptom, viz., extreme coldness of that side of the body which is to become paralyzed after a time, just the reverse of what will occur when the paralysis is complete, owing to a spasmodic contraction of bloodvessels preceding their paralysis and dilatation.

"I remember on one occasion, after one of my lectures at the Royal College of Surgeons in London, that eminent physician, Dr. T. Addison (whose modesty, like that of almost all truly great men, was in proportion to his great talents and extensive learning), did me the great honor of asking my opinion upon a case in which the symptoms were, besides the extreme coldness, already mentioned, in one half of the body, some tingling in the fingers, a very slight ptosis of the external rectus, some jerks in the external muscles of the face, on the side opposite to that of the injury (this is a symptom you do not find when the injury is higher up than the pons Varolii), also some sensations of tickling in the face (another symptom you do not find in cases where the injury is higher up than the pons); in fact the symptoms were such that although, as observed in the human species, they were new to me, who was at that time more of a physiologist than a practitioner, I had no hesitation, simply from the teachings of physiology, in pronouncing the case to be one of disease of the pons Varolii; and so it proved, as it gradually and successively presented all the symptoms which I have mentioned as characteristic of disease of that organ. As I had not, however, the advantage of making an autopsy of the case, you might think me very presumptuous in holding that I had made a certain diagnosis; but really with this class doubt is impossible, when the symptoms are combined, forming a group so definite and distinct that there is absolute certainty, even during life, as to their cause. It is not so when the disease goes higher up in the brain; we are then often at a loss, and it is extremely difficult to say, even, whether there is organic disease or mere temporary disorder of the circulation, and still less is it possible to say what part of the brain is the seat of the disease.

"I pass on to the consideration of another kind of hemiplegia. There is one kind of that paralysis perfectly distinct from all those which I have mentioned. We will suppose a patient comes to you with some slight stiffness and tendency to throw his limbs in a wrong way when he walks. There is not a very great paralysis, but rather a decided weakness, and hardly any loss of sensibility on one side of the body. He complains also of noises in the ear on that side, of feeling extremely giddy, and of having sometimes a tendency to turn round upon himself, like a top. Sometimes he reels as if he were intoxicated. He very frequently finds it impossible to walk straight forward. Sometimes, also, he has very great hyperæsthesia to sounds; at times, also, he has a sudden tendency to fall down—it seems to him as if he cannot keep up, and that he must fall; also, that if he takes hold of something he will keep up.

"This class of cases is, indeed, one of the most instructive of all kinds of hemiplegia. I have now collected more than twenty-two such cases—not all seen by me. According to the autopsy which has been made in a number of these cases, they are simply instances of reflex paralysis, not paralysis arising from the alteration or destruction, or, in fact, from any interruption of the conductors of voluntary motion—the paralysis in these cases is due to quite another cause. In this class of paralysis there is disease either of the petrous bone or of the base of the brain near the origin of the fifth pair of nerves, or near the place of entrance of the auditory nerve. There is, in this case, not a destruction but an irritation by a pressure (and not a very considerable pressure) on the crus cerebelli, or a small part of the pons Varolii, or the medulla oblongata. Place a tumor there which has encroached slightly and gradually on the parts of the base of the brain I have named, and the symptoms I have mentioned will appear. But let the injury go further, and the paralysis on one side of the body, viz., the side where the injury exists, will disappear; and yet the injury to the brain is greater now than it was before. From the moment that a real disorganization has taken place in the base of the brain the symptoms which existed at first disappear, and the paralysis passes from the right side of the body to the left side—the tumor being at the right side.

"I regret that I cannot, owing to the limited time at my command, explain the causes of this at length; but I shall endeavor to do so in a few words. At first, in a case of the kind I speak of, there is an irritation starting from the injured part or parts, acting in the same way to produce a paralysis or an irritation from a nerve in any part of the body which causes a reflex paralysis. Acting upon the brain, it produces, by a reflex influence, an alteration of some kind which the microscope has, as yet, been unable to detect, and owing to which is due a paralysis. But why is it that, if the disease at the base of the brain progresses, the paralysis disappears on the side first affected and appears on the opposite side? The explanation is this: The part which, in the first place, was irritated, has now been destroyed altogether; there is no more irritation, and the paralysis consequently ceases on that side, but it passes to the other side of the body, because in the pons Varolii and medulla oblongata there are conductors of voluntary motion passing above their decussation to go up to the brain; and hence, if an injury takes place such as to destroy some of these conductors, there will be paralysis on the opposite side of the body.

"I intended bringing forward several other types of hemiplegia to show the great assistance physiology affords in explanation of such cases, but I am compelled to be brief. I must, however, mention one other species of hemiplegia—that due to hemorrhage in the cerebellum. In this case there are features which lead to accurate diagnosis; and many of these features have been discovered by experiments upon animals. One of these symptoms is vomiting; this is a constant feature of hemorrhage in the cerebellum. There may be also hyperæsthesia in some parts of the body—not the whole body, or even half, but in some portions. There will often likewise be amaurosis; this is generally due, not to pressure on the tubercula quadrigemina, as has been stated, but to a reflex action, as in the majority of cases there is no pressure whatever upon the tubercula quadrigemina. That it is due to a reflex action appears still more clearly when we consider what occurs in many of these cases; we may find the amaurosis existing in the left eye alone, in the right eye alone, or in both eyes—nay, more, we may find amaurosis passing alternately from one eye to the other in the same patient, although in all those cases the disease is only in one-half of the cerebellum, showing, in fact, that there is no persistency or uniformity of action in the production of amaurosis in these cases; or, in other words, that there is the variety we know to exist in effects due to a reflex influence.

"There is another kind of hemiplegia as to which I must say a few words—I mean that which is due to a lesion of the anterior lobes of the brain. Phrenologists, you know, have regarded the anterior lobes as the organs of speech; but there have been many instances—Dr. Stokes mentioned a very remarkable one to me a few days ago—in which there has been destruction of these parts without any deprivation of speech. But the question remains (and it is an interesting one), what occasions the loss of speech when such loss takes place? As regards this question I shall, in a moment or two, have to point out how great a variety of symptoms may be produced by a lesion of almost every part of the brain. The deprivation of speech I hold to be a reflex phenomenon; and that it is so, we have almost a proof in the fact tnat it often varies very much in the same patient, according to circumstances which physiology has, as yet, been unable to detect, but certainly with the lesion of the brain still continuing unaltered. It is worthy of remark, too, that the loss of speech is usually unaccompanied by any loss of movement in the tongue; there may be perfect freedom of motion in the tongue, and the deprivation of speech arises from the circumstance of the patient being *unable to give expression to his thoughts;* and this inability extends not merely to speech, he is equally powerless to express ideas either by signs or by writing. The paralysis, in fact, is a 'paralysis of the organ of expression of ideas;' and it is remarkable that this occurs, while the individual may remain, in other respects, in full possession of his intellectual faculties, at least so far as we can judge of this possession. One of the cases of that kind I have seen was that of a clergyman, a man of remarkable intelligence. He had not lost the mechanical part of the power of speech, for he articulated a few words very distinctly—but they were sounds devoid of meaning; he was equally unable to express his thoughts by writing, or even by signs. Even when he was told to express 'yes' by lifting up one finger, and 'no' by lifting up two fingers, he was unable to do it, and showed signs of great distress at his inability; and all this, although he appeared, in other respects, very intelligent.

"I pass on to notice another form of disease showing the importance of a knowledge of physiology. It is a form to the discovery of which I have been partly led by experiments on animals. A patient may come to you complaining of pain in the back, of a pricking sensation in both arms, with some degree of itching, burning, or some subjective sensation of alternate cold and heat, or some curious variety of cutaneous eruption, differing from those you have usually to deal with when they are not due to nervous disease. You may find, also, some weakness or even a great paralysis in the two upper limbs; jerkings in those limbs, likewise a stiffness in some of the muscles, and tenderness under pressure. If you do not know the physiological meaning of all these symptoms, you will be led to suppose that there is some local affection—rheumatism, perhaps, of both the arms. You will think it very strange that you can find no description of any such disease in books—yet the explanation of all these phenomena, when read by the light of physiology, is very simple. The symptoms depend altogether on an inflammation of the nerves of the arms at or before their exit from the spine in the lower cervical region and the upper dorsal region. The neuritis is usually accompanied by a local spinal meningitis; all the symptoms arise from irritation of the motor and sensitive nerves, and also of the nerves of bloodvessels. If, then, you meet such a case as I have described, and applying your physiological knowledge you arrive at the true character of the injury, you may, by adopting a certain course of treatment, cure, or, at all events, greatly mitigate the disease. I have met many cases of the kind; and, with the exception of one, which I saw in consultation with Mr. W. Adams, of North London, and which terminated in death, all the cases have been either cured completely or more or less ameliorated. The treatment consists in a most active blistering of the spine in the region of the disease; also, in the application of dry cupping. Injections of narcotics have also been resorted to; but I repeat that the principal part of the treatment consists in the repeated application of blisters to the spine. Internally I have also employed iodide of potassium; but what share it has had in the cure I do not know.

"I intended to have brought forward many varieties of cases of disease of the spinal cord, but time does not allow of my doing so. I will, however, say that physiology has demonstrated these most important facts—that the spinal cord, in its most central part, which is decidedly insensible and inexcitable in its normal state, may become exquisitively sensitive and excitable under the influence of inflammation or congestion, and that when the gray matter has become sensitive, it may occasion all those strange sensations and other symptoms complained of by patients attacked with either myelitis, or great congestion of the gray matter—viz., pricking as by pins and needles, formication, itching, feeling of heavy pressure, or tightness, coldness, or heat, &c., and jerks, trembling, cramps, convulsions, contraction, &c. In cases of paraplegia this group of symptoms is generally due to a special alteration in the condition of the spinal cord, and the part of this group concerning sensation cannot be produced without congestion or inflammation, and characterize essentially these two diseases, especially the latter; and if you do not find them in cases of paraplegia, you may be convinced that the spinal cord is free from congestion, and still more free from inflammation.

"Keeping in view the object of this lecture, viz., the importance of a deep knowledge of physiology in the practice of medicine and surgery, I now proceed to notice the distinct and almost opposite features of two cases of fracture of the spine in the cervical region. Two patients, I suppose, are brought to you—each having sustained an injury of that portion of the spine, and having, in consequence, a complete paralysis of the trunk and limbs. One of them is almost pulseless, extremely cold, and covered with a clammy perspiration. He appears almost like a dead man; there is no contraction of the muscles, the limbs hang loose and dead, his breathing almost gone, his pulse—not only very faint, but extremely slow—from 40 to 45 in a minute. If you commit the fault of bleeding him, you find that the venous blood flows out red like arterial blood—flows out, not with a great impulse, for the heart is very weak and almost in a state of syncope, but still it flows out not like venous blood—it has an impulse like arterial blood.

"Now examine tbe other patient. In him the symptoms are almost the exact contrary to those in the former. The limbs are stiff and rigid; the pulse unnaturally high; the heart's action much excited; the heat of the body, not only higher than is usual in the limbs, but absolutely higher, and by many degrees, than the temperature of the blood in health in man. If you bleed him, which may prove useful, you find that the venous blood is darker than usual, and comes out without any trace of impulse.

"Now what is the explanation of these two cases? How is it that one of these patients is in a state of syncope, and the other in a state of asphyxia? It is found, by experiments performed upon animals, that in the former of these two cases the cause is an irritation—perhaps extremely slight, for the slightest prick may be sufficient—of the spinal cord, the effect of which is a stoppage of the heart's action, so that it beats with diminished force and rapidity, and as a consequence of this condition of the heart all the other symptoms above described ensue. In the other patient, on the contrary, the spinal cord has actually been cut across altogether, and the patient is in a far *worse* state, in reality, though he *seems* to be far more alive than the other. He *seems* to have the power of reaction which we wish to find in patients, yet the danger of his state is far greater—in fact, he is sure to die, while the other, by means of an operation (trephining, or resection of the spine, an operation which was performed by Dr. R. M'Donnell, to-day, upon a patient in this city), may possibly survive.

"I proceed now to make some remarks on symptoms of brain disease. As you are aware, our view of the symptoms of disease of the brain proper—i. e., the cerebral lobes—is, that an injury there produces paralysis by striking the organ of the will, that there is a paralysis of the will at least for that part of the body which is paralyzed, and that if other symptoms occur, they are due also to a loss of function of the part altered in the brain, or to pressure upon neighboring parts. I have not time fully to demonstrate that the symptoms of brain disease are generally due to a reflex action, but I shall show (I hope satisfactorily) that the admitted view is absolutely untenable. You are all familiar with the great variety of symptoms presented in brain disease. Take, for instance, facial paralysis. In cases of disease of the brain, paralysis (as you know) does not exist in the orbicularis, but in the other muscles of the face. Now if it is alleged that when, in cases of disease of the anterior lobe of the brain, for instance, there is facial paralysis, it is because the nerve fibres of the facial nerve go to that part. I am perfectly willing to admit it; but let us take a case of injury to a different part of the brain—say, for instance, an injury in the posterior lobe—how will you explain facial paralysis in this case? Do the nerve fibres of the facial nerve go to the anterior lobe in one case, and to the posterior in the other? Surely not. In fact, the consequence of such a hypothesis would be that there is absolutely no part of the brain which would not be the spot to which the nerve fibres of the facial nerve go. If you can suppose such a hypothesis possible, I ask you to reconcile the facts with what anatomy teaches—viz.: that the facial nerve goes to a certain part of the pons Varolii, and no higher; so that besides the absurdity of supposing that the facial nerve goes to every part of the brain, and each part containing all these fibres, there is likewise the anatomical impossibility which arises when we examine the course of the facial nerve. Again, take another instance. The tongue, as you are aware, is in most of these cases of disease of the cerebral lobes, more or less paralyzed; there is some difficulty in drawing it out in a straight line—also some slight impediment in the speech, owing to paralysis of some of the fibres of the ninth pair of nerves. How will you reconcile the existence of that paralysis in the majority of cases, with the fact that we do not see the fibres of the ninth pair—the hypoglossal nerves—going up higher than the medulla oblongata? Here is a patient who has a complete destruction of one-half of the pons Varolii; mark, that that organ must be the place of passage—if there is any such passage—of the fibres of the hypoglossal nerves going up to the brain. Therefore, when one-half of the pons is diseased, there must be a paralysis of the tongue; yet in most cases of disease of the pons there is no paralysis of the tongue, so that both anatomy and this clinical fact prove that the hypoglossal nerves do not go to the cerebral lobes. How, then, you will explain the fact that disease in any part of these lobes may (as we know, by experience, it does) produce paralysis of the hypoglossal pair of nerves, I leave you to decide. To me it seems clear, that to hold that when an injury to any portion of the cerebral lobes causes paralysis of the ninth pair, or of any other nerve, it is because the paralyzed nerves go to that part of the brain, is decidedly wrong. If you examine a number of cases of brain disease, especially of an acute character, such as cases of quickly enlarging tumors producing irritation, and especially tumors pressing on the dura mater, you will find that for a tumor in one and the same part of the brain there is, in some cases, no symptom at all produced, and in other cases you may find any symptom whatever. I do not think you could point out any feature of nervous complaint that you will not find existing in some one or other of the recorded cases of injury to, or disease of, any particular part of the brain. Nay more, even in one and the same individual, with one and the same persistent disease, you will often have a great change in the symptoms. He may be paralyzed to-day, another day he may not; you may, in fact, have every variety of phenomena, or no phenomenon at all, all arising from one and the same cause; so that unless you go the length of supposing that each individual part of the brain possesses every function whatever, and has the effect of acting on every part of the body in a direct way, you cannot explain these facts. Mark, that in some cases of injury to, or disease of, the cerebral lobes, there are no phenomena at all; so that, while on one hand, you are driven to suppose that there is no part of the brain which does not contain all the nerves of the body, you are, on the other hand, forced to conclude that there is no part of any nerve of the body going to the cerebral lobes. Such a hypothesis is obviously impossible. I have time only to say that the explanation which I have ventured to offer of these phenomena is, that they come under the class of effects produced by reflex action.

"I shall now pass to quite different cases, but also due to a reflex action, and I will first speak of syncope when induced by a blow on the stomach. Experiments on animals have shown me that in these cases the syncope is produced by a reflex action through the abdominal sympathetic ganglions, the spinal cord, and medulla oblongata, and, at last, the par vagum. I have often and often tried the experiment on animals, of crushing the ganglions of the sympathetic in the abdomen. In such cases there was sometimes a sudden arrest of the heart's action, in other cases only a temporary diminution in the beating of the heart, while in still other cases there was hardly any effect produced. In those animals in which the effect on the heart was produced, I waited till recovery was established. I then divided the par vagum, after which I crushed again the ganglions; not the least effect was then produced on the heart's action, clearly showing that the transmission takes place through the par vagum. In those cases in which the heart is stopped, whether from the cause above assigned, or any other, such as drinking very cold water when in perspiration, or an emotion, etc.,—in those cases of reflex syncope when the patients are on the verge of death, and would almost surely die if nothing were done for their immediate relief—in such conditions there is one means of restoring life, which I have found by experiments on animals to be of the utmost importance, and so much so that very frequently, even when the action of the heart was quite stopped, I have been able by simply pressing on the sternum, and by giving a hard push to the heart, to make it beat again; and after repetition of the same means to make it resume its action. It will not beat long if the cause of the syncope is a powerful one, but still it will beat; and if you continue the use of the means mentioned, it will continue to beat, and in that way you may succeed in reviving a patient. But this is not all. If you add to that cause of revival another, which is most powerful, and which is directly the reverse of what John Hunter tried upon himself when he found he was a state of syncope one day at the College of Surgeons—if nstead of making the patient breathe as quickly as he can you stop his breathing altogether, just as if you were trying to kill him by suffocation, you revive him. By producing a state of asphyxia for about half a minute, the patient may be saved; he will have a struggle, and come out of it very quickly. Nothing, indeed, is more powerful to make the heart beat than an accumulation of carbonic acid in the blood. Whether I have been right or wrong in maintaining, as I have done, that the normal and abnormal beatings of the heart, when very tumultuous, depend chiefly upon an irritation by the carbonic acid of the blood—whether I am right in this respect or not—there is no question that if you produce temporary suffocation in these cases, you make the heart beat again, and beat with force. I should add that I have not the merit of having discovered this fact, as I find that in an old book, entitled the *Surgeon's Mate*, and published more than 200 years ago, a writer of the name of Woodall has mentioned it as very important. He, however, does not say on what he grounded his view. There are some other features about syncope of great importance. In any case where the circulation is impeded, you may in a moment throw one or even two pounds of blood into the trunk and head, by simply pressing hard on the four main arteries of the body. If you press those four main arteries, you prevent the circulation going on in them, and at once a considerable quantity of blood returns from the venous system to the trunk, especially if the limbs are kept up so as to allow gravitation to help the movement of the blood.

"A few words now upon asphyxia. There are experiments which show, as clearly as possible, that if you take two animals, one of them having had the temperature of its body very much diminished, the other at a normal temperature, and if you dip them both into water at the same time, the one which has had its temperature reduced will survive twice, three times, and sometimes even five times as long as the other—the duration of life under water being sometimes extended to twelve or fifteen minutes. The greater the previous lowering of temperature, the longer the duration of life There is another very interesting fact. It is well known that persons who have fallen into water have, in many cases, been drawn out and revived after an immersion of a number of minutes. Now, in experiments performed upon animals by applying galvanism to the par vagum so as to stop the heart's action—which is an effect that a fall into water will sometimes produce, by a reflex action from the influence of cold on the skin, or by an emotion—we find life will last much longer; in other words, the animal will be able to survive a much longer time when dipped under water, from having had an attack of syncope just before. This case, then, is exactly the reverse of the former. In one, syncope is cured by asphyxia; in the other, asphyxia is rendered less mortal because syncope previously existed. I need not stop to show that all these notions we owe to physiological science.

"I shall now say a word upon poisoning. Poisoning often produces death by causing such a diminution of temperature as is incompatible with life. Take, for example, two animals which have been poisoned with opium. Supposing the temperature to be cold in the room, lay one of them near a fire and covered carefully with warm clothes, and let the other be exposed to the cold in a corner far from the fire. You will find, *cæteris paribus*, that the one which is kept warm will survive, while the other will die. This fact we find with almost every organic poison, viz., that there is considerable diminution of temperature produced—if not, *per se*, sufficient to occasion death, enough, at any rate, to add a powerful cause to the other causes existing. Now this diminution of temperature is a feature which we can fight against; and it is, therefore, of the utmost importance, in cases of poisoning, to use every means to keep up the temperature of the body."—*Dublin Quart. Journ. Med. Sci.*, May, 1865.

